# Trace Elements in Crustaceans, Mollusks and Fish in the Kenyan Part of Lake Victoria: Bioaccumulation, Bioindication and Health Risk Analysis

**DOI:** 10.1007/s00244-020-00715-0

**Published:** 2020-02-04

**Authors:** James Omondi Outa, Chrispin O. Kowenje, Annemariè Avenant-Oldewage, Franz Jirsa

**Affiliations:** 1 Department of Limnology and Bio-Oceanography, University of Vienna, Althanstrasse 14, 1090 Vienna, Austria; 2 Department of Chemistry, Maseno University, P.O. Box 333, Maseno, 40105 Kenya; 3 Institute of Inorganic Chemistry, University of Vienna, Waehringer Strasse 42, 1090 Vienna, Austria; 4 Department of Zoology, University of Johannesburg, P.O. Box 524, Auckland Park, 2006 South Africa

## Abstract

This is the first comprehensive report on the accumulation of Cr, Ni, As, and Ag in the fish species Nile tilapia *Oreochromis niloticus* and Nile perch *Lates niloticus* from Lake Victoria, complemented with recent data on Cu, Zn, Cd, and Pb. This also is the first report on Cr, Ni, As, and Ag levels in invertebrates: the shrimp *Caridina nilotica*, gastropod *Pila ovata*, and bivalve *Mutela bourguignati*. The study was conducted at five sites in the Kenyan part of Lake Victoria: four sites in Winam Gulf influenced by various anthropogenic pressures, including a site near Kisumu City, and one in the main lake, with lesser direct anthropogenic influence. Apart from Cu and Ag, which were highest in *O. niloticus* liver, the invertebrates had higher levels of trace elements than fish. Contamination of the gulf with trace elements was best mirrored by the invertebrates, whose mobility is limited; they accumulated Cr, Cd, Ag, and Pb corresponding to the levels in the surface sediment. The accumulation of trace elements in fish species and their bioindicative potential corresponded to their habitats and feeding behaviour. The tissue contents of most trace elements were higher in the inshore-dwelling, omnivorous *O*. *niloticus* compared to the pelagic, piscivorous *L*. *niloticus*. Cu (465 ± 689 mg/kg dw) and Ag (3.45 ± 1.49 mg/kg dw) in the liver of *O*. *niloticus* were up to 10 and 119 times higher than in *L*. *niloticus*, respectively. *Oreochromis niloticus* therefore has bioindicative potential for Cu and Ag contamination. Both the invertebrates and fish showed positive correlations between Cu and Ag concentrations, indicating similar source and/or uptake route. The target hazard quotients (THQ) show that there is no human health risk associated with the consumption of these fish. However, the levels of Zn, Cd, and Pb in *P. ovata* surpassed maximum food safety limits and are hence potentially unsafe for human consumption.

**Electronic supplementary material:**

The online version of this article (10.1007/s00244-020-00715-0) contains supplementary material, which is available to authorized users.

Trace elements, which include heavy metals and metalloids, occur naturally in the environment (Biney et al. 1994). Their multiple industrial, domestic, agricultural, medical, and technological applications have led to their wide distribution in the environment, raising concerns over their potential effects on human health and the environment (Tchounwou et al. 2012). Trace elements pose a threat to wildlife and humans when occurring in elevated concentrations (Luczynska et al. 2018). According to the report by Pure Earth and Green Cross (2016), five of the top six global pollutants are heavy metals (Cr, Cd, Hg, and Pb) and the metalloid (As), from which at least 200 million people worldwide are at risk from toxic pollution at levels above international health standards.

Aquatic ecosystems consist of several compartments, each of which has certain physicochemical properties that determine the fate of trace elements therein. In sediments, water, and biota, trace elements show different partitioning according to the properties of these elements, as well as the physicochemical and biological processes that influence their deposition, sorption, and remobilization between the sediment and the water column (Mwamburi 2013). Aquatic biotas are subject to the full range of chemical and physical influences of their habitat and therefore are sound indicators for the status of the respective aquatic systems (Chovanec et al. 2003). For instance, aquatic invertebrates take up and accumulate essential and nonessential trace elements from their environment through permeable body surfaces and from their diet. The result is an enormous variability in tissue and body concentrations of different elements in different taxa (Rainbow 2007). Parmar et al. (2016) noted that macroinvertebrates, such as crustaceans that live near the bottom of water bodies, have restricted mobility and therefore are powerful indicators of ecological conditions. Mollusks have been established as useful bioindicator tools, because their metal body burden often reflects the concentrations of metals in the surrounding water and sediment (Gundacker 2000; Hayes et al. 2015). Fish also have attracted much attention in the biomonitoring of surface water pollution. This reflects not only their special biological characteristics, such as relatively large body size and long-life cycle, but also the health risk to fish consumers (Zhou et al. 2008). Plessl et al. (2017) reported that fish are valuable bioindicators for evaluating trace element pollution when the life habits of the species are considered. Ecological and toxicological studies mostly use the gills, liver, kidney, and muscles tissue of fish, with muscles (edible part) being the most appropriate in terms of human health risk analysis (Luczynska et al. 2018). Aquatic biota play an important role in biomonitoring, and the main purpose of measuring trace elements concentration in this biota is to determine the toxicological threat to humans from ingesting excessive element loads in edible species (Depledge et al. 1998). The toxicity of heavy metals and metalloids has promoted various efforts around the world to set limits in food products from aquatic ecosystems. For instance, the European Union (EU) and World Health Organisation (WHO) have set maximum levels for As, Cd, Pb, and Hg as priority substances for consumable aquatic products (EU 2008; FAO/WHO 2017). In addition, the United States Environmental Protection Agency (USEPA) has developed the target hazard quotients (THQ) formula, which is applicable in assessing the risk faced by people exposed to elements, such as Cr, Fe, Ni, Cu, and Zn, through the consumption of contaminated fish (USEPA 2018a). THQ is the ratio between the potential exposure to a substance and the reference dose (level at which no adverse effects are expected) (USEPA 2018a). Nonetheless, the problem of toxic pollution remains prevalent in many low- and middle-income countries in many parts of the world (Pure Earth and Green Cross 2016).

Inland waters and freshwater biodiversity constitute a valuable natural resource in economic, cultural, aesthetic, scientific, and educational terms (Dudgeon et al. 2006). Lake Victoria, the largest lake in Africa, is the most important freshwater resource for the local population (Crul 1995), and an estimated 30 million people are dependent on the lake in one way or another. Mbabazi and Wasswa (2010) noted that industrial, agricultural, and domestic waste discharge have increased the levels of heavy metals in the lake, putting aquatic organisms and human consumers at risk. Moreover, contamination is prominent in large embayments of the lake, notably Winam Gulf (Kavirondo/Nyanza Gulf), Murchison Bay, and Mwanza Gulf, which are influenced by industrial and municipal wastes from rivers and the adjacent cities (Njuru et al. 2013; Outa et al. 2019). Various studies have investigated the levels of trace elements in the lake’s surface sediments and macrophytes, e.g., Kishe-Machumu and Machiwa (2003), Ochieng et al. (2006), Mwamburi (2013), and Outa et al. (2019). In contrast, very few studies have examined the accumulation of trace elements in the fauna; most of these have focused on Hg and a few selected trace elements in the muscle tissue of fish. The earliest study is probably from 1987 by Wandiga and Onyari (Biney et al. 1994) on Mn, Fe, Cu, Zn, Cd, and Pb in fish muscle tissue. Other studies include Campbell et al. (2003), who reported on Hg in the African catfish *Clarias gariepinus*, Nile tilapia *Oreochromis niloticus*, and Nile perch *Lates niloticus*, and Mbabazi and Wasswa (2010), who investigated the contamination of the silver cyprinid *Rastrineobola argentea* with Cu, Zn, Cd, and Pb. More recently, Ongeri et al. (2012) reported on the accumulation of Fe, Cu, Zn, Cd, and Pb in the muscle tissue of *O. niloticus* and *L. niloticus* and whole *R. argentea* samples taken between 2006 and 2007 from Winam Gulf.

This means that very scarce information is available on the bioaccumulation of other trace elements, such as Ni and As, and virtually no records of Cr and Ag in the fauna of the lake. Apart from Mwamburi (2013), who reported on Pb accumulation in the crustacean *Caridina nilotica* and on Fe, Mn, Cu, and Pb in the bivalve *Sphaerium* sp., there are no other reported studies on trace element contents of invertebrates, which form an integral part of the aquatic food chain. In a recent study, Outa et al. (2019) reported that the surface sediment at Winam Gulf was contaminated with Cr, Cu, Zn, Ag, and Pb at levels that surpassed the consensus-based threshold effect concentrations (TEC) sediment quality guidelines outlined in Macdonald et al. (2000) and that the sediments at the inshore part of the gulf near Kisumu City were severely polluted with Cu and Pb. Ag and Pb have no established biological function, whereas Cr, Cu, and Zn are essential but with a narrow range of concentrations between beneficial and toxic effects (Tchounwou et al. 2012). Unlike most heavy metals whose natural occurrence, anthropogenic release into the environment and toxicity have been known for decades, Ag has become a new potential pollutant in recent years (McGillicuddy et al. 2017; Plessl et al. 2019). According to WHO (2002), emissions from smelting operations as well as from the manufacture and disposal of certain photographic and electrical supplies were some of the anthropogenic sources of silver in the biosphere in the past. Recent studies have shown an increase in the usage of silver nano-particles in numerous consumer products, such as textiles, medical products, domestic appliances, food containers, cosmetics, paints, and nano-functionalised plastics in many parts of the world (McGillicuddy et al. 2017).

In the view of these findings, we performed a comprehensive study on the accumulation of trace elements in the lake’s fauna with a link to potential health risks posed to humans who consume food products from the lake. This study targeted native invertebrate species: the decapod crustacean *C*. *nilotica* (Atyidae), the gastropod *Pila ovata* (Ampullaridae), the bivalve *Mutela bourguignati* (Iridinidae), and commercial fish: *O. niloticus* (Cichlidae) and *L. niloticus* (Laditae). *Caridina nilotica* is the only shrimp species known in Lake Victoria (Fryer 1960). Its littoral populations are epibenthic detritivores, while offshore populations may engage in facultative planktivory (Lehman et al. 1996). This shrimp is an important feed component for fish and other animals for small-scale and commercial farming in East Africa (Bundi et al. 2013; Mwamburi 2013). *Mutela bourguignati* is a sedentary filter feeder (Graf and Cummings 2006) and is mainly used as bait for fish. *Pila ovata* predominantly inhabits shallow inshore waters and feeds on a variety of items, such as biofilms, periphyton, macrophytes, other invertebrates, and carrion (Hayes et al. 2015). Like most apple snails, it has become a focus of research following its introduction to many parts of the world as a source of food protein or as aquarium pets (Hayes et al. 2015); it is regarded as an important food protein source for human populations in Congo and around Lake Victoria (Van Damme 2017). *Oreochromis niloticus* is an inshore fish species and an opportunistic herbivore that feeds on algae, small invertebrates, and detritus (Njiru et al. 2004). *Lates niloticus* juveniles are inshore dwellers and feed mainly on crustaceans and insect larvae but gradually become pelagic and piscivorous as they grow to maturity (Kitchell et al. 1997). *Lates niloticus* and *O*. *niloticus*, introduced into the lake in the 1950s, are two of the three major commercial fish species (the other being *R. argentea*) (Budeba and Cowx 2007). According to FAO (2015), despite Kenya having 142 400 km^2^ of marine water, the inland Lake Victoria contributes approximately 75% (approx. 139,500 tons) of Kenya’s total aquatic food production. Moreover, 35% of the lake’s total fish production is landed in Kenya for internal and external markets, of which Nile perch *L. niloticus* accounts for 84.7% of total fish exports to Europe, Asia, United States, and Central America (FAO 2015).

In summary, this study investigated eight trace elements (Cr, Ni, Cu, Zn, As, Ag, Cd, Pb) in the invertebrates *C. nilotica*, *P. ovata*, and *M*. *bourguignati* and the commercial fish species *O. niloticus* and *L. niloticus* with respect to bioaccumulation, bioindicative aspects, and the potential health risk to consumers.

## Materials and Methods

### Study Area Descriptions

**Fig. 1:**
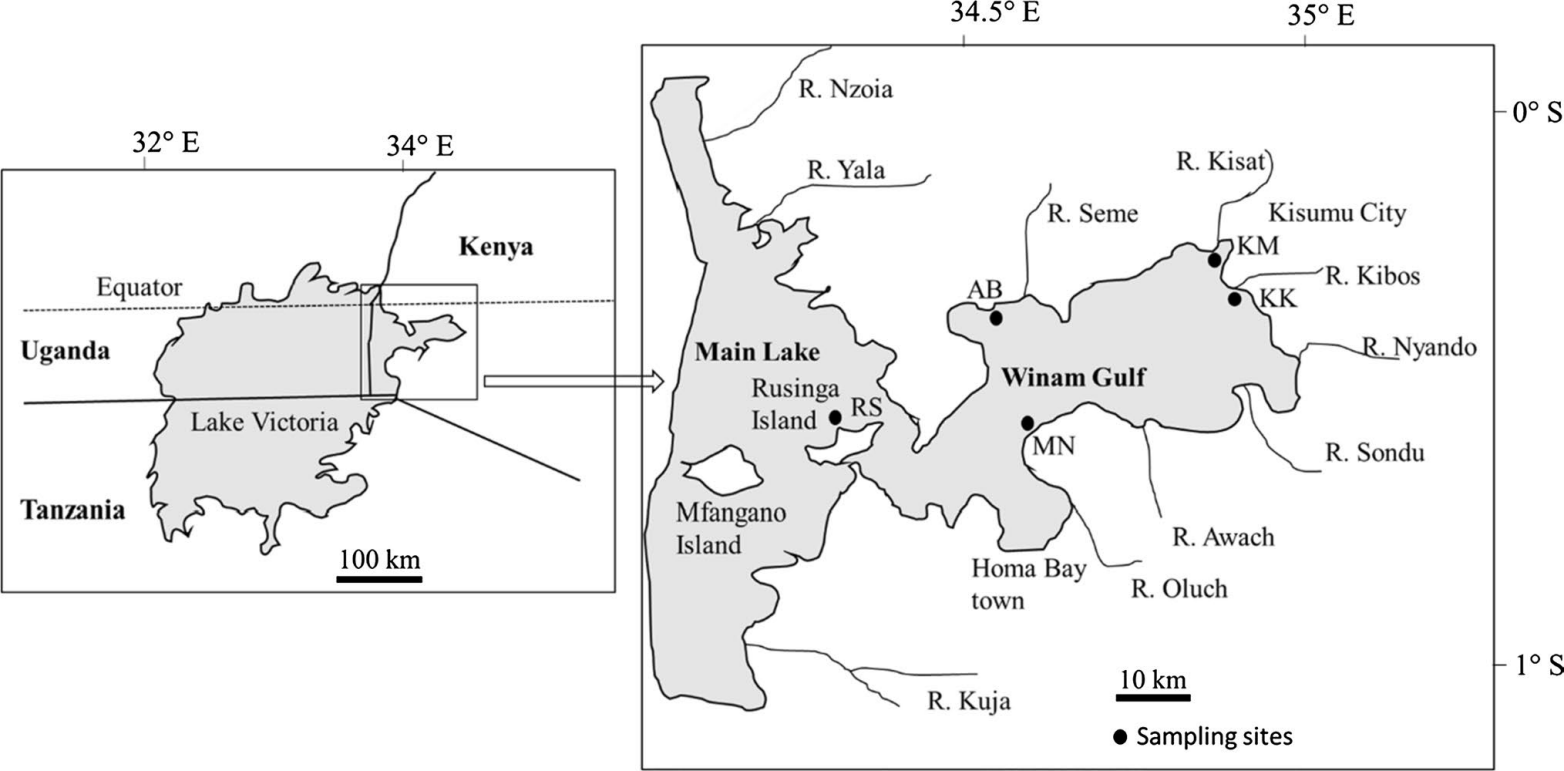
Map of Lake Victoria indicating the study area and sampling sites. Modified from Okungu et al. (2005). AB: Asembo Bay (0° 11′ 10.2″ S 34° 23′ 35.8″ E), KM: Kisumu City (0° 05′ 16.4″ S 34° 44′ 59.0″ E), KK: Kisumu City outskirt (0° 09′ 41.4″ S 34° 44′ 51.6″ E), MN: Mainuga (0° 20′ 48.7″ S 34° 29′ 09.1″ E), and RS: Rusinga Island (0° 23′ 20.5″ S 34° 11′ 48.9″ E)

Lake Victoria, shared by Kenya (6%), Uganda (43%), and Tanzania (51%), is the world’s largest tropical lake and the second largest freshwater lake in the world, covering a total of 69,000 km^2^ with a mean depth of 40 m and maximum depth of 79 m (Okungu et al. 2005). It is located along the equator between 0.5° N and 2.5° S and 32° E and 34° E at an elevation of 1134 m above sea level, with a large catchment of 195,000 km^2^. The main river inlet (Kagera) drains through Rwanda, Burundi, Tanzania, and Uganda, while the main river outlet is the Nile (Crul 1995). The Kenyan portion of Lake Victoria lies just south of the equator between 0° 6′ S to 0° 32′ S and 34° 13′ E to 34° 52′ E, and it covers an area of approximately 4200 km^2^ of which 1400 km^2^ comprises the Winam Gulf, (Lung’ayia et al. 2001). Although Kenya’s share of the lake is only 6%, it contributes significantly to internal and external fish markets (FAO 2015). The Winam Gulf (Fig. 1) is a shallow basin with an inshore (mean 4 m, maximum 6 m depth) and offshore zone (mean 12 m, maximum 43 m depth) (Crul 1995). The gulf receives high river inflows from western Kenya highlands (Njuru and Hecky 2005). The Lake Victoria basin has an equatorial climate, with temperatures ranging between 20 and 35 °C, and the mean annual rainfall ranges between 1000 and 1500 mm (Okungu et al. 2005).

The study was performed in five selected sites in the Kenyan part of Lake Victoria both in Winam Gulf and part of the main lake as indicated in Fig. 1. The sampling sites: Asembo Bay (AB), Kisumu City (KM), Kisumu City outskirt (KK), Mainuga (MN), and Rusinga Island (RS) were selected based on the different types and levels of anthropogenic pressures. As noted in Outa et al. (2019), contamination of the water and surface sediment was highest at KM, which is a part of the lake that suffers from the effects of urbanization, with industrial and municipal wastewater discharges from Kisumu City. AB, located on the northern shoreline, and MN to the south, within the mid-region of Winam Gulf, are moderately contaminated by runoff and deposition of sediment from rural farmlands. In contrast, RS is part of the main lake body with minimal horizontal mixing with the gulf and represents the characteristics of the main lake body in this study.

### Sampling and Sample Preparation

Sampling in the lake was conducted between January and July 2017. *Caridina nilotica* was obtained from AB, KM, and RS using seine nets. The samples were transferred into clean plastic bags, put on ice, and were transported protected from light to Maseno University laboratory in Kisumu City, where they were rinsed with Millipore water. In order to achieve sufficient material for analyses, 15–20 specimens were pooled into 15-ml polypropylene (PP) tubes and oven-dried for 48 h to constant weight at 90 °C. In total, 72 sample were obtained: 26 from AB, 24 from KM, and 22 from RS. Mollusc specimens of *P. ovata* (≈ 40-mm shell height) and *M. bourguignati* (≈ 60-mm shell length) were handpicked (wearing nitrile gloves) from the littoral areas of the study sites. The specimens were transported alive to the laboratory in aerated containers with lake water. For *P. ovata*; 10 specimens from AB, 17 from KM, 7 from MN, and 15 from RS were taken, whereas for *M. bourguignati* 8 specimens were obtained from MN and RS each. The specimens were kept in aerated lake water for 48 h for depuration (Gundacker 2000; Chapman 2016): after depuration, the animals were rinsed with Millipore water, individually put into plastic bottles and killed by freezing them at − 20 °C; they were stored in the freezer until further processing. After thawing the mollusks, the soft body was separated from the shells using a ceramic knife and plastic tweezers. For *P. ovata*, the visceral mass and foot were placed into separate PP tubes, whereas in *M. bourguignati* the entire soft body was used. The samples were oven dried for 48 h to constant weight at 90 °C. Fish were caught using gill nets from AB, KM, KK, MN, and RS and transported alive to the laboratory in aerated tanks with lake water. In total, 63 specimens of *O. niloticus* and 46 specimens of *L. niloticus* were obtained. The fish were killed by cervical dislocation and dissected. Tissue samples, approximately 5 g of dorso-caudal muscle and the same mass of liver, were obtained from each fish using a ceramic knife and plastic tweezers, placed into PP tubes, and dried for 48 h to constant weight at 90 °C. The samples were shipped to the University of Vienna, Institute of Inorganic Chemistry laboratory for further processing and chemical analysis.

### Sample Analyses

Dried tissue samples were homogenized with a mortar and pestle, and approximately 0.2 g was digested in 9 ml of 34% HNO_3_ (TraceSELECT^®^ Fluka) and 1 ml of 30% H_2_O_2_ using a microwave MARS XPRESS system (CEM Corporation). The digested samples were transferred into 15-ml volumetric flasks and brought up to volume using milliQ water. The leachates were filtered through 0.2-µm PTFE pre-syringe filters (VWR). Reference samples comprising 0.2 g (dry weight) of fish protein (DORM-3 and DOLT-5) obtained from the National Research Council Canada (NRCC) were digested and diluted in the same manner as described above. Trace element (Cr, Ni, Cu, Zn, As, Ag, Cd, Pb) concentrations were determined using total x-ray reflection fluorescence spectrometry (S2 PicoFox TXRF, Bruker) and, when necessary, graphite furnace atomic absorption spectrometry (GF-AAS) using a PinAAcle 900Z (Pelkin Elmer).

### Statistical Analyses

Statistical analyses were done using IBM SPSS 21. The Shapiro–Wilk Test, which is very sensitive to detecting deviations from normality (Mohd and Yap 2011), was used to check for the normality of data distribution. The differences in trace element content of the invertebrates and fish were determined by comparing site-specific means of each species using parametric one-way ANOVA and nonparametric Kruskal–Wallis and Mann–Whitney *U* tests (for pairwise comparisons) where applicable. For the ANOVA post hoc test, Tukey’s HSD was applied for pairwise mean comparisons where homogeneity of variance was established, whereas the Games Howell test was applied for unequal sample sizes and variances (Games and Howell 1976; Kim 2015). In each of the sampling sites, intraspecific differences in trace element accumulation and partitioning in the foot and viscera of mollusks and in muscle and liver tissue of fish were compared using the parametric Student’s *t* test and nonparametric Mann–Whitney *U* test where appropriate. All differences were tested for significance at *p* < 0.05. The correlations between trace elements concentrations in the invertebrates and fish were tested for significance using the parametric Pearson’s correlation test and nonparametric Spearman’s rank correlation test where applicable. Similarly, we tested the correlations between element concentrations in muscle and liver tissue in relation with total length (TL) of fish.

The bioaccumulation factors (BAFs) were determined according to the formula:

$$ {\text{BAF}}_{{\left( {\frac{x}{y}} \right)}} = \frac{{{\text{Concentration}}\;{\text{of}}\;{\text{element}}\;{\text{in}}\;x}}{{{\text{Concentration}}\;{\text{of}}\;{\text{element}}\;{\text{in}}\;y}}. $$

In which the variables x and y stand for matrices that are compared to each other, such as sediment, invertebrate, and fish tissue. Element concentrations in the sediment were based on data from Outa et al. (2019).

### Risk Assessment

The body trace element burden (mg/kg dw) of mollusks and fish muscle obtained in our study were converted to fresh weight by applying a conversion factor of 0.2 based on an estimated water content of 80% (Ongeri et al. 2012; Heuzé and Tran 2017). The trace element content of mollusks was compared with existing maximum limits for food safety (EU 2008; CFDA 2014; FAO/WHO 2017). The target hazard quotients (THQ) were determined for trace elements to assess the risk to people who consume *O. niloticus* and *L. niloticus* in the region. THQ is the ratio between the potential exposure to a substance and the reference dose (level at which no adverse effects are expected) (USEPA 2018a). THQ values > 1 were regarded to pose a risk to human fish consumers in relation to a specific element, and THQ ≤ 1 meant no health risk. A THQ of 0.1 was later suggested for noncarcinogenic pollutants to consider additive effects (USEPA 2018a). For calculation, the standard equation inputs for fish consumption given by USEPA were used, with the exception of body weight, to reflect the local realities. USEPA uses a body weight (Bwa) of 70 kg, whereas the average body weight in Africa is 60.7 kg (Walpole et al. 2012), which we used. The equation used to determine THQ (USEPA 2018b) is as follows:

$$ {\text{THQ}} = \frac{{{\text{EF }} \times {\text{ED }} \times {\text{IRF }} \times {\text{C}}}}{{{\text{RfDo }} \times {\text{BWa }} \times {\text{AT}}}} $$

The variables in the equation are defined as follows: EF is the exposure frequency (350 days/year); ED is the exposure duration (26 years); IRF is the average fish consumption per day. The per capita fish consumption in Kenya is 4.5 kg per year (FAO 2015), which translates to 0.0123 kg/day. C is the trace element concentration in the edible portion of fish (element muscle content, mg/kg ww); RfDo is the oral reference dose (mg/kg/day) according to regional screening levels (USEPA 2018c); Bwa is the average body weight (60.7 kg); and AT is the average time for noncarcinogens $({\text{AT}} = 365\;{\text{days }} \times {\text{ED}} = 9490\;{\text{days}}$).

## Results and Discussion

Recovery rates for elemental analyses in reference samples are shown in Table 1. The total lengths and weights of the fish specimens are given in SM Table 1. Due to the high mobility of *L*. *niloticus*, we treated all fish for this study as one sample, whereas values for *O. niloticus* were calculated per site. With the exception of Cu and Ag, which were highest in *O*. *niloticus* liver, the invertebrates had the highest concentrations of the trace elements measured in this study. The levels of most trace elements were higher in the liver than in the muscle of fish (Tables 2, 3, 4). In freshwater fish, the liver stores and detoxifies various trace elements, hence accumulating them to higher levels compared with muscle tissue (Coetzee et al. 2002; Luczynska et al. 2018; Plessl et al. 2019). Similarly, the levels of most trace elements in invertebrates were higher in the viscera than the foot. These results agree with various studies on mollusks (Gundacker 2000; Deng et al. 2008; Bao et al. 2018). Relatively low concentrations of trace elements in other mollusk soft body parts, such as the foot and head, indicate that the snails have developed mechanisms to regulate the translocation of trace elements from the viscera (Deng et al. 2008). Regarding human consumption, calculated THQs for Cr, Ni, Cu, and Zn in fish muscle were below 0.1; the consumption of these fish therefore poses no health risk to humans. In the following, each of the elements are briefly discussed separately:

**Table 1:** Recovery rates for the analyses of certified reference material (NRCC) for the respective methods

Element	Method	Reference material	Reference value (mg/kg)	Measured value (mg/kg)	Recovery rate (%)
Cr	GF-AAS	DORM-3	1.89 ± 0.17	1.89 ± 0.11	99.3
Ni	GF-AAS	DOLT-3	2.72 ± 0.35	3.05 ± 0.18	108.7
Cu	TXRF	DOLT-5	35 ± 2.4	34.1 ± 0.8	97.3
Zn	TXRF	DOLT-5	105.3 ± 5.4	107.2 ± 5.5	101.8
As	GF-AAS	DOLT-5	34.6 ± 2.4	30.3 ± 3.7	87.6
Ag	GF-AAS	DOLT-3	1.2 ± 0.07	1.19 ± 0.28	99.5
Cd	GF-AAS	DOLT-3	19.4 ± 0.6	19.7 ± 0.5	101.5
Pb	GF-AAS	DOLT-3	0.32 ± 0.05	0.33 ± 0.01	104.2

**Table 2:** Mean concentrations (± SD) of essential trace elements (mg/kg dw) in invertebrates from the study sites

Species	Site	*n*	Cr	Ni	Cu	Zn
*C. niloticus*	AB	26	3.27^a^ ± 3.0	2.57^a^ ± 2.0	148^a^ ± 12	69.8^a^ ± 7.6
	KM	24	3.58^a^ ± 3.2	1.26^b^ ± 1.5	115^be^ ± 11	72.8^a^ ± 23
	RS	22	1.19^be^ ± 1.0	0.791^c^ ± 0.69	77.4^c^ ± 7.1	72.6^a^ ± 7.0
*P. ovata* foot	AB	10	0.501^c^ ± 1.1	1.45^ab^ ± 1.9	73.1^b^ ± 26	65.7^ac^ ± 8.6
	KM	17	0.607^c^ ± 1.0	1.72^b^ ± 3.2	108^be^ ± 71	54.3^b^ ± 8.8
	MN	7	0.271^d^ ± 0.19	0.325^c^ ± 0.2	94.2^be^ ± 80	49.7^b^ ± 5.1
	RS	15	0.570^c^ ± 0.92	0.879^c^ ± 2.1	112^bc^ ± 79	61.4^c^ ± 19
*P. ovata* viscera	AB	10	5.05^a^ ± 2.0	36.1^d^ ± 13	57.8^d^ ± 13	1350^d^ ± 433
	KM	17	3.75^a^ ± 2.7	12.3^e^ ± 9.9	135^e^ ± 66	893^de^ ± 769
	MN	7	2.13^e^ ± 1.4	20.1^f^ ± 7.8	105^be^ ± 71	638^e^ ± 336
	RS	15	2.41^e^ ± 1.9	9.63^e^ ± 3.4	90.3^bc^ ± 83	1361^d^ ± 1132
*M. bourguignati*	MN	8	3.72^a^ ± 1.4	4.85^a^ ± 2.4	9.15^f^ ± 1.6	829^e^ ± 460
	RS	8	3.71^a^ ± 3.5	1.32^b^ ± 1.0	6.45 ^g^ ± 1.9	383^f^ ± 176

Mean values followed by same letters for each element do not differ significantly (*p* > 0.05)

**Table 3:** Mean concentration (± SD) of nonessential trace elements (mg/kg dw) in invertebrates from the study sites

Species	Site	*n*	As	Ag	Cd	Pb
*C. niloticus*	AB	26	0.828^a^ ± 0.13	0.212^a^ ± 0.03	0.069^a^ ± 0.01	4.57^a^ ± 3.6
	KM	24	0.858^a^ ± 0.15	0.422^c^ ± 0.04	0.067^a^ ± 0.01	4.71^a^ ± 2.7
	RS	22	0.957^b^ ± 0.06	0.096^b^ ± 0.01	0.046^b^ ± 0.005	4.38^a^ ± 2.3
*P. ovata* foot	AB	10	0.277^c^ ± 0.09	0.255^a^ ± 0.09	0.178^ah^ ± 0.09	0.282^b^ ± 0.34
	KM	17	0.717^a^ ± 0.43	0.660^d^ ± 0.25	0.060^ab^ ± 0.03	0.735^d^ ± 0.76
	MN	7	0.401^c^ ± 0.10	0.300^a^ ± 0.27	0.035^bc^ ± 0.03	0.159^b^ ± 0.06
	RS	15	0.371^c^ ± 0.20	0.354^a^ ± 0.23	0.026^c^ ± 0.02	0.201^b^ ± 0.33
*P. ovata* viscera	AB	10	0.497^d^ ± 0.13	0.148^b^ ± 0.05	5.57^d^ ± 3.03	1.86^c^ ± 1.4
	KM	17	0.623^ad^ ± 0.27	0.711^d^ ± 0.29	2.51^e^ ± 2.87	15.5^e^ ± 30
	MN	7	0.366^c^ ± 0.11	0.356^a^ ± 0.43	1.32^e^ ± 0.52	1.56^c^ ± 0.48
	RS	15	0.195^e^ ± 0.06	0.272^e^ ± 0.17	0.599^f^ ± 0.42	0.479^d^ ± 0.40
*M. bourguignati*	MN	8	0.123^e^ ± 0.05	0.035^f^ ± 0.02	0.463 ^g^ ± 0.21	1.42^c^ ± 0.68
	RS	8	0.091^e^ ± 0.04	0.021^f^ ± 0.01	0.274 ^h^ ± 0.20	0.736^b^ ± 0.97

Mean values followed by same letters for each element do not differ significantly (*p* > 0.05)

**Table 4:** Mean concentration (± SD) of trace elements in muscle and liver (mg/kg dw) of fish in the study area: site specific for *O*. *niloticus* and pooled samples for *L*. *niloticus*

Species	Site	Tissue	*n*	Cr	Ni	Cu	Zn	Ag	Cd	Pb
*O. niloticus*	AB	Muscle	9	0.131^a^ ± 0.08	0.047^a^ ± 0.03	0.652^a^ ± 0.21	23.3^a^ ± 6.3	< 0.015	< 0.005	0.158^a^ ± 0.12
		Liver	9	0.304^b^ ± 0.39	0.187^b^ ± 0.02	240 ^cd^ ± 66	130 ^cd^ ± 12	0.584^a^ ± 0.11	0.062^a^ ± 0.01	0.494^b^ ± 0.15
	KM	Muscle	20	0.119^a^ ± 0.08	< 0.040	2.12^b^ ± 1.7	23.0^a^ ± 4.2	< 0.015	< 0.005	0.129^a^ ± 0.11
		Liver	20	0.371^b^ ± 0.50	0.190^b^ ± 0.08	320 ^cd^ ± 231	98.4^c^ ± 18	3.45^b^ ± 1.49	0.197^b^ ± 0.21	0.552^b^ ± 0.32
	KK	Muscle	9	0.127^a^ ± 0.09	< 0.040	0.766^a^ ± 0.16	17.2^b^ ± 2.7	< 0.015 ±	< 0.005	0.123^a^ ± 0.11
		Liver	9	0.379^b^ ± 0.30	0.188^b^ ± 0.05	465^c^ ± 689	127 ^cd^ ± 15	0.640^a^ ± 0.55	0.067^a^ ± 0.08	0.486^b^ ± 0.14
	MN	Muscle	6	0.360^ab^ ± 0.63	< 0.040	0.826^a^ ± 0.37	23.2^a^ ± 6.3	< 0.015	< 0.005	0.085^a^ ± 0.03
		Liver	6	0.252^ab^ ± 0.19	0.180^b^ ± 0.03	178^d^ ± 74	89.6^c^ ± 7.7	0.584^a^ ± 0.31	0.058^a^ ± 0.03	0.083^a^ ± 0.02
	RS	Muscle	20	0.102^a^ ± 0.06	0.072^a^ ± 0.03	0.720^a^ ± 0.35	19.4^ab^ ± 4.5	< 0.015	< 0.005	0.108^a^ ± 0.08
		Liver	20	0.367^b^ ± 0.35	0.166^b^ ± 0.07	170^d^ ± 123	87.5^c^ ± 10	0.657^a^ ± 0.50	0.333^c^ ± 0.27	0.125^a^ ± 0.06
*L. niloticus*		Muscle	46	0.150^a^ ± 0.17	0.095^c^ ± 0.03	1.08^a^ ± 0.22	18.3^ab^ ± 3.2	< 0.015	< 0.005	0.088^a^ ± 0.09
		Liver	46	0.303^b^ ± 0.28	0.149^b^ ± 0.09	46.8^e^ ± 15.8	157^d^ ± 30	0.032^c^ ± 0.02	0.067^a^ ± 0.05	0.413^b^ ± 0.16

Values followed by same letters for each element do not differ significantly (*p* > 0.05)

### Cr

The essentiality of chromium for plants and animals is still under discussion (Markert et al. 2015), but it is a well-known pollutant of aquatic environments worldwide (Pure Earth and Green Cross 2016). As indicated in Table 2, Cr concentrations in the *C*. *nilotica* and *P*. *ovata* viscera showed distinct spatial variation (one-way ANOVA, *F* = 5.476, *p* = 0.006 and *F* = 3.827, *p* = 0.016 respectively), which we did not observe in *P. ovata* foot or in *M*. *bourguignati*. *Caridina nilotica* from the less disturbed RS site had lower concentrations than samples from the AB and KM sites (Games Howell test, *p* = 0.006 and 0.005, respectively). Similarly, *P*. *ovata* viscera from RS had lower concentrations compared with AB and KM (Tukey HSD test, *p* = 0.020 and 0.046, respectively). This agrees with the results for Cr in sediments from these sampling sites (Outa et al. 2019), where concentrations were significantly lower at RS. These findings indicate that *C*. *nilotica* and *P. ovata* viscera are potentially good indicators for Cr contamination, because their metal content corresponds with environmental concentrations. There are very few studies on Cr accumulation for invertebrates and virtually no records of Cr levels in *C*. *nilotica, P*. *ovata*, and *M*. *bourguignati*. Tu et al. (2008) reported Cr mean body concentrations ranging from 0.167 to 0.633 mg/kg dw in the caridean river prawn *Macrobrachium rosenbergii* (Palaemonidae) from the Mekong River Delta, Vietnam. This is even lower than the mean Cr concentration recorded in *C. nilotica* from the RS site (1.19 ± 1.0 mg/kg dw). Regarding human consumption, most international regulations do not provide maximum values for Cr. One exception is China, where a maximum level of 2 mg/kg ww is allowed in mollusks (CFDA 2014). All levels from our study were below this, and therefore no risks for consumers are expected.

Chromium concentrations in fish liver were significantly higher than in muscle in all samples, and there was no significant difference between the two fish species (Table 4). Comparing the sample sites, no significant differences were recorded either in muscle or liver (Kruskal–Wallis test, *X*^2^(4) = 2.263, *p* = 0.688 and *X*^2^(4) = 2.073, *p* = 0.722, respectively). In fish, Cr is predominantly taken up through the gills and distributed via the blood to the tissues, with little or no bioconcentration at environmentally relevant concentrations (Reid 2012). As noted by Outa et al. (2019), the levels of dissolved Cr in Lake Victoria water were generally low (mean: 0.14–0.39 µg/l), and this may be the main reason for the low values recorded in fish tissue. Contrary to the findings of Coetzee et al. (2002), who recorded positive correlations between Cr tissue content and size of omnivorous *Clarias gariepinus* and the benthic feeder *Labeo umbratus*, fish size did not correlate to tissue Cr concentrations. Because no data have been published on the accumulation of Cr in fish from Lake Victoria, we compared our findings to those from other freshwater bodies in the region. Plessl et al. (2017) reported mean Cr concentrations of 0.42 ± 0.33 and 1.65 ± 1.24 mg/kg dw in muscle and liver, respectively, in *Tilapia zillii* from Lake Naivasha (Kenya), which are higher than in the fish species from our study.

### Ni

Very few studies on Ni accumulation in invertebrates are available, and no records on Ni concentrations in *C*. *nilotica, P*. *ovata*, or *M*. *bourguignati*. In the current study, the variations in concentrations in crustacean and mollusc samples from the study sites (Table 2) did not correspond with data on the sediments at those sites as reported in Outa et al. (2019). *Pila ovata* viscera had the highest concentrations (9.63–36.1 mg/kg dw), which were at least four times higher than in the other invertebrate tissues. Compared with other studies, lower Ni levels have been reported in mollusks: 0.02–1.54 mg/kg dw in *Pomacea caniliculata* from the Mae Klong River, Thailand (Peña, 2017) and a mean concentration of 5 mg/kg dw in the viscera of the bivalve *Unio pictorum* from Lake Maggiore, Italy (Ravera et al. 2003). Because the levels in Lake Victoria water and sediment were generally low, except for the MN site (Outa et al. 2019), the high levels of Ni in *P*. *ovata* viscera from our study warrant further investigation.

In fish, Ni can be taken up through the gills or olfactory epithelium during waterborne exposures or through the gut during dietary exposures. It preferentially accumulates in the kidneys (Pyle and Couture 2012). Higher concentrations were recorded in the muscle of *L. niloticus* than in *O. niloticus* (Table 4), which might be due to differences in physiological regulation in the two fish species. Moderate negative correlations occurred between TL and Ni concentration in *L. niloticus* liver (Spearman’s rank test, *r*_s_ = − 0.432, *p* < 0.01). This agrees with Coetzee et al. (2002), who noted that the Ni content of fish was predominantly negatively correlated with fish length. The Ni content of *O*. *niloticus* in our study did not correspond to its concentration in the environment, possibly due to effective physiological regulation. Other studies report different results; Mwamburi (2013) did not detect Ni in the muscle tissue of three fish species (*O. leucostictus*, *T. zillii*, *Micropterus salmoides*) from Lake Naivasha, Kenya. In our study, the value was below the LOD (0.040 mg/kg dw) in muscle tissue of *O. niloticus* in three of the sampling sites (KM, KK, MN) but was detected in the rest of the samples, albeit at low levels. In the rather unpolluted Vaal Dam in South Africa, similar levels were reported from cyprinid fish (Gilbert et al. 2017). Much higher Ni concentrations were reported from the polluted Olifants Rivers System (South Africa): 14.2 and 19.4 mg/kg dw in muscle tissue, and 16.6 and 26.1 mg/kg dw in liver tissue of *C*. *gariepinus* and *L*. *umbratus*, respectively (Coetzee et al. 2002).

### Cu

Cu is an essential element whose levels in tissues are well regulated in invertebrates (Depledge et al. 1998; Rainbow 2007) and fish (Grossel 2012). Data from mollusks and crustaceans did not reflect the sediment pollution levels reported at KM (Outa et al. (2019)). The Cu concentrations in the foot and viscera of *P. ovata* did not differ significantly (Mann–Whitney test, *U* = 1141, *p* = 0. 672), indicating that translocation to various organs, including the foot is not restricted. *Pila ovata* accumulated 10–18 times more Cu than *M. bourguignati*. This finding concurs with Gundacker (2000), who reported that freshwater unionid bivalves had extremely low Cu contents compared with their gastropod counterparts in urban river habitats in Austria. This variation was attributed to different regulation capacities for Cu by the mollusc taxa. As indicated in SM Table 2, the BAF values for Cu in the invertebrates in relation to sediment were > 1: the highest BAFs were 5.26 (*C*. *nilotica*), 4.40 (*P*. *ovata* viscera), and 3.96 (*P*. *ovata* foot). Comparing Cu levels with other studies, Abdennour et al. (2000) reported mean range values of 44.5–123 mg/kg dw in the shrimp *Atyaephyra desmarestii* at four sites in Algeria, which is comparable with values for *C*. *nilotica* (77.4–148 mg/kg dw). In mollusks, lower levels were recorded in snails and bivalves from less contaminated freshwater bodies. For instance, in the Ipoba Stream, Nigeria, Ezemonye et al. (2006) recorded Cu concentrations ranging from 2.0 to 5.8 mg/kg dw in the viscera of *P. ovata*, whereas mean values of 2.1 and 4.5 mg/kg dw were recorded in the bivalve *M. spekei* from two sites in Lake Tanganyika, Tanzania (Foxall et al. 2000). The bivalve *M. bourguignati* samples from the two sites in our study had higher levels: 6.45 ± 1.6 and 9.15 ± 1.9 mg/kg dw from MN and RS, respectively. At the same time, the *P*. *ovata* viscera values from our sites are in the same magnitude as data on *Pomacea canaliculata* viscera (60–170 mg/kg dw) from the polluted Fankou stream in southern China. In fish, Cu is predominantly taken up from the diet through the intestines and directly from the water across the gill epithelia (Grossel 2012). The levels in the muscle of *O. niloticus* at the KM site were higher than at all other sites (Table 4), corresponding with the Cu pollution in the sediment at KM. Accordingly, *O*. *niloticus* muscle tissue is a potential indicator for Cu contamination. This also indicates that physiological Cu regulation in fish may break down in individuals living in habitats polluted with Cu. At all sampling sites, the liver values of *O*. *niloticus* were higher than the corresponding levels in the surface sediment (SM; Table 2). The levels in *O*. *niloticus* liver were 4–10 times higher than in the liver of *L. niloticus*. The omnivorous inshore-dwelling *O. niloticus* is probably exposed to more Cu uptake through the diet and from contact with the contaminated water and surface sediment. Cu in fish tissue generally shows weak or negative correlations with fish size (Szarek-Gwiazda and Amirowicz 2006; Luczynska et al. 2018). That also was the case for the Cu concentration in the muscle tissue of *L*. *niloticus*: values showed a moderate negative correlation with TL (Spearman’s rank test, *r*_s_ = − 0.456, *p* < 0.0001). Nonetheless, the Cu content in *O. niloticus* liver had high positive correlations with TL (Spearman’s rank test, *r*_s_ = 0.785, *p* < 0.0001) at KM, where the Cu levels were highest in surface sediment. This agrees with the findings of Çoğun et al. (2003), who recorded high positive correlations between *O. niloticus* size and liver content in Cu exposure experiments. Compared with other studies, the Cu levels in the muscle tissue of both fish species and in the liver of *L*. *niloticus* are of the same magnitude as in the same fish species from Winam Gulf (Ongeri et al. 2012) and from Lake Nasser, Egypt (Mohamed 2008). The mean concentrations of Cu in *O. niloticus* liver (170–465 mg/kg dw) from our study sites, however, were higher than in the same species from Lake Nasser (67.1–102 mg/kg dw) (Mohamed 2008) and also higher than in *T. zillii* (123 ± 148 mg/kg dw) from Lake Naivasha (Plessl et al. 2017). The higher Cu values in *O*. *niloticus* liver from our study reflect the higher levels in the study area.

### Zn

The body concentration of Zn, an essential element, is regulated to relatively constant levels in invertebrates (Depledge et al. 1998; Rainbow 2007) and freshwater fish (Hogstrand 2012). In our study, elevated levels of Zn at KM (Outa et al. 2019) were not reflected in the fauna. This might indicate that the Zn in the invertebrates and fish were within their internal regulation capacities. As indicated for SM in Table 2, BAF values for Zn in the *P*. *ovata* viscera and *M*. b*ourguignati* in relation to sediment levels were > 1; the highest BAFs were 16.5 (*P*. *ovata* viscera) and 7.89 (*M*. *bourguignati*).

Compared with other studies, Abdennour et al. (2000) reported Zn mean range values of 37–73 mg/kg dw in the atyid shrimp *A*. *desmarestii* in Algeria (comparable to the 69.8–72.9 mg/kg dw in *C. nilotica* from our study stites). The mean concentrations in *M*. *bourguignati* and *P. ovata* viscera from our study sites (383–1361 mg/kg dw) are higher than in molluscs from other areas in Africa. For instance, mean Zn concentrations in the viscera of *P*. *ovata* from various sites in the Ikpoba River in Nigeria ranged from 23.6 to 45.2 mg/kg dw (Ezemonye et al. 2006). Mwamburi (2013) recorded a mean concentration of 207 mg/kg in *Sphaerium* sp. from Lake Victoria, whereas Foxall et al. (2000) reported means of 72 and 76 mg/kg dw in *M. spekei* from Lake Tanganyika, Tanzania. Importantly, Deng et al. (2008) reported much higher levels in the viscera of *Pomacea canaliculata* in Fankou stream (southern China), which received effluent from a Zn/Pb mine. There, mean Zn concentrations in *P. canaliculata* ranged from 3000 to 6400 mg/kg. Regarding the risk for consumption by humans, the mean levels of Zn in *P*. *ovata* viscera at AB (1350 ± 433 mg/kg dw), KM (893 ± 769 mg/kg dw), and RS (1361 ± 1132 mg/kg dw) and *M*. *bourguignati* at MN (829 ± 460 mg/kg dw) surpassed various national food safety limits: Australian Legal Requirements for Food Safety (750 mg/kg dw), Ministry of Public Health, Thailand (667 mg/kg dw), and Brazilian Ministry of Health (250 mg/kg dw) (Yap et al. 2004). According to Hogstrand (2012), due to slower metabolism in older fish, Zn accumulation and fish size are either negatively correlated or lack any significant relationship. Our study also indicates negative correlation between Zn content in muscle tissue and TL of fish: *L. niloticus* (Spearman’s rank test, *r*_s_ = − 0.317, *p* = 0.032) and *O. niloticus* (Spearman’s rank test, *r*_s_ = − 0.453, *p* = 0.045). Elsewhere, mean Zn values of 12.1 mg/kg and 10.3 mg/kg were recorded in the muscle tissue of *O. niloticus* and *L. niloticus*, respectively, from Lake Nasser (Mohamed 2008) – at least twofold lower than in our study. Very high concentrations were reported in muscle tissue of *O. leucostictus* (604 ± 82 mg/kg dw) from Lake Naivasha (Otachi et al. 2014) and for *Hydrocynus forskahlii* (426 ± 215 mg/kg dw) from Lake Turkana, Kenya (Plessl et al. 2017). Those studies attributed the high levels of Zn in fish muscle to elevated Cd levels, which block Zn containing enzymes, enhancing their expression. In liver tissue, Zn concentrations in *L*. *niloticus* 157 ± 30 mg/kg dw and mean concentrations in *O*. *niloticus* (87.5–130 mg/kg dw) from our study were higher than the mean concentrations in *O. leucostictus* (73.1 mg/kg dw) (Otachi et al. 2014), in *O. niloticus* (40.8 mg/kg dw) and in *L. niloticus* (37.8 mg/kg dw) from Lake Nasser (Mohamed 2008).

### As

Very few studies are available on As accumulation in freshwater invertebrates, and there are no records for *C*. *nilotica, P*. *ovata*, and *M*. *bourguignati*. The As concentrations in the invertebrates and fish were very low in this study. Arsenic has no known biological role, and accumulation in freshwater biota is mostly through direct uptake from water, generally only at environmentally elevated levels (Mcintyre and Linton 2012). In our study area, the concentrations were generally low in water (1.47–1.89 µg/l) and surface sediment (1.19–6.19 mg/kg dw) (Outa et al. 2019). This may help to explain the low levels in the fauna. The mean levels in *C. nilotica* from our study sites (0.828–0.957 mg/kg dw) were on the same magnitude with the findings of Tu et al. (2008) who recorded mean range concentrations of 0.41–1.46 mg/kg dw in *M*. *rosenbergii* from the Mekong River Delta, Vietnam. The mean concentrations in molluscs at our study sites (0.195–1.41 mg/kg dw) were lower than in the bivalve *Unio pictorum* from Lake Maggiore, Italy (12 mg/kg dw; Ravera et al. 2003). The content in both *P. ovata* and *M. bourguignati* was below the maximum limits for human consumption (0.5 mg/kg ww) given by the China Food and Drug Administration (CFDA 2014). In fish, As was not detected in any muscle samples (LOD = 0.02 mg/kg dw). In fish liver tissue, it was detected in *O. niloticus* specimens from RS (0.036 ± 0.01 mg/kg dw), and the mean concentration in *L. niloticus* was (0.043 ± 0.01 mg/kg). Our results agree in part with the findings of Machiwa (2003), who did not detect As (LOD = 0.01 µg/g ww) in any liver and muscle samples of *O. niloticus* and *L. niloticus* from the Tanzanian side of Lake Victoria. In contrast, Ngure et al. (2014) recorded mean range values of 0.015–1.92 mg/kg As in whole samples of the silver cyprinid *R. argentea* from four sites in a gold mining region around Lake Victoria. This contrasting finding points to the possibility of interspecific and regional differences arising from anthropogenic impacts.

### Ag

**Fig. 2:**
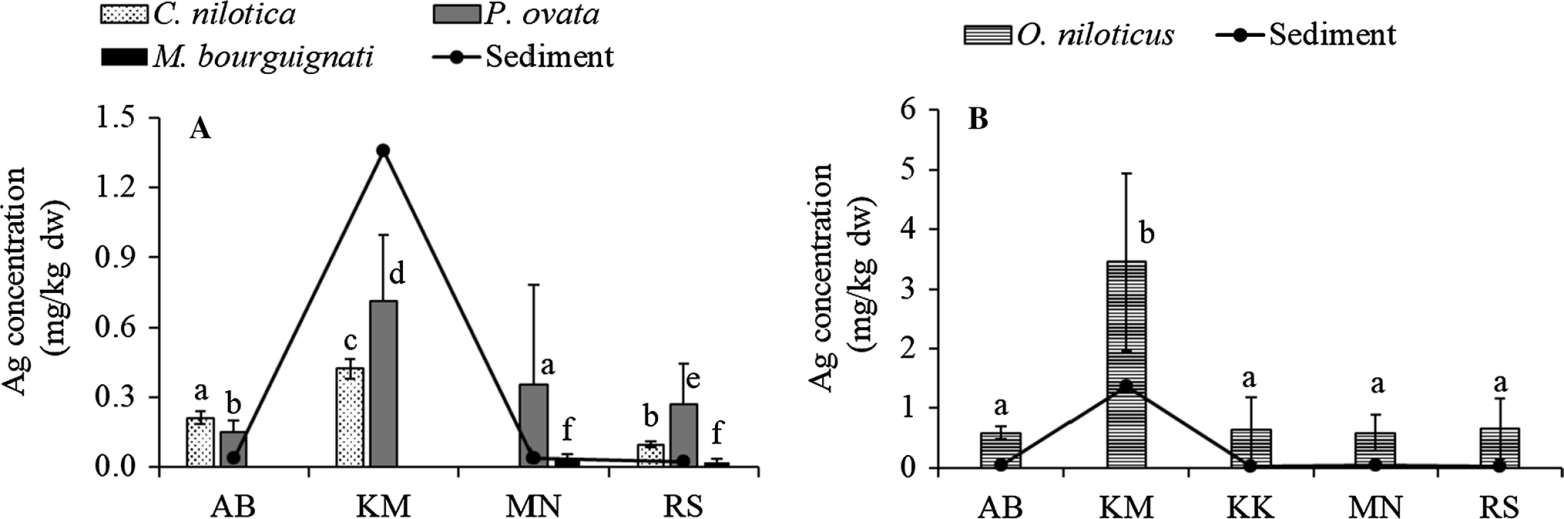
Concentration patterns of Ag in *C. nilotica*, *P. ovata* viscera and *M. bourguignati* (**a**) and *O. niloticus liver* (**b**) in the current study and the corresponding surface sediment content in different sampling sites (Outa et al. 2019)

**Fig. 3:**
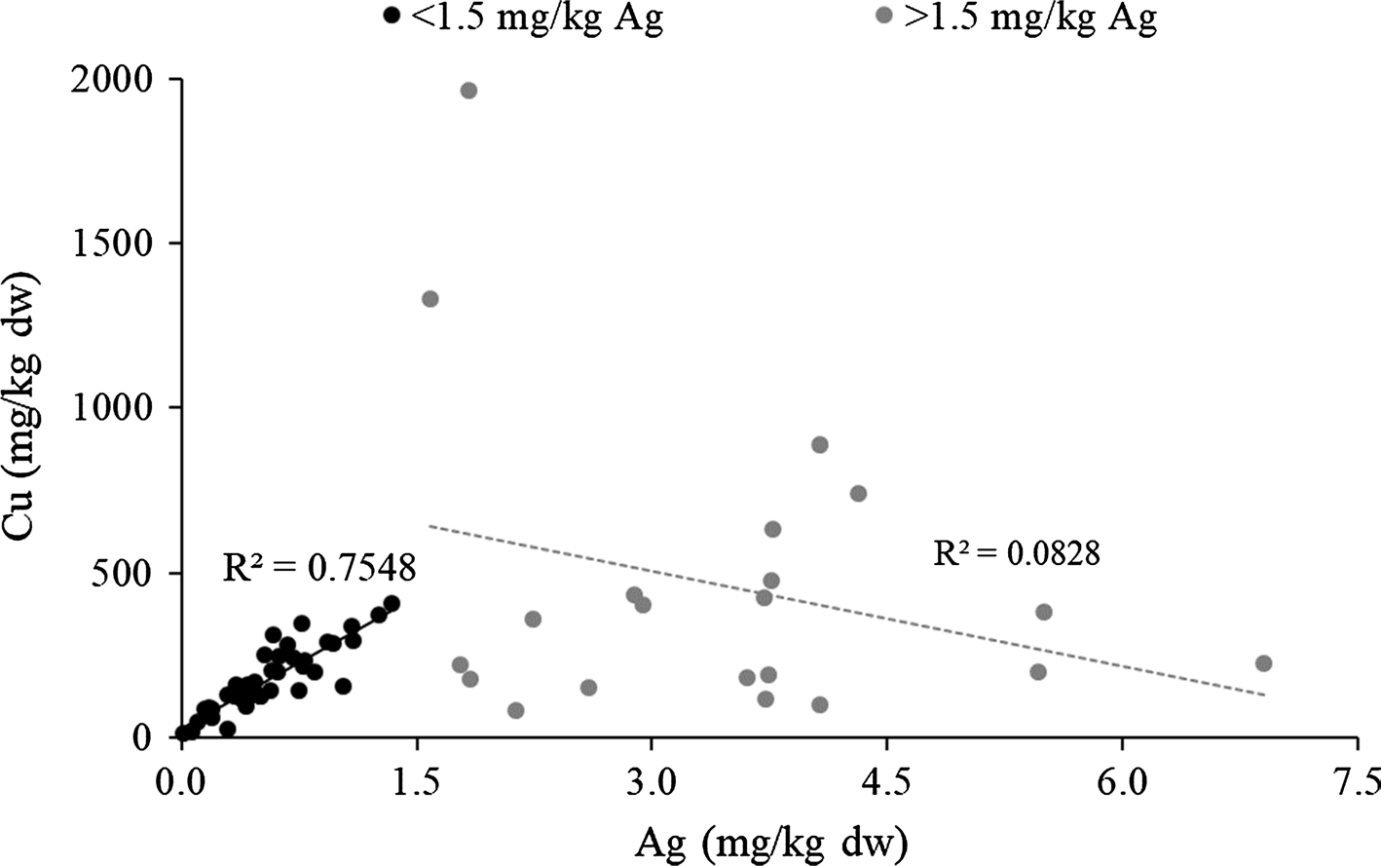
Correlation between Cu and Ag concentrations in *O. niloticus* liver

Ag is accidentally taken up by aquatic animals through Na and/or Cu transport pathways, and animals from waters with low Ag levels can have substantial levels of Ag in their tissues, raising the question whether Ag could be a micronutrient (Wood 2012). There are no previous studies on the Ag levels in the fauna of Lake Victoria. The concentrations in *C*. *nilotica* and *P*. *ovata* foot and viscera varied significantly between the study sites (one-way ANOVA; *F* (2, 69) = 688.030, *p* < 0.001, *F* (3, 45) = 9.425, *p* < 0.0001 and *F* (3, 45) = 13.286, *p* < 0.0001). As indicated in Fig. 2, levels in the invertebrates corresponded with surface sediment concentrations; *C*. *nilotica* and *P*. *ovata* are therefore potential indicators for Ag contamination. Like with Cu and As, the levels in the foot and viscera of *P. ovata* did not differ significantly (Table 3). The translocation of Ag within the soft tissue of this snail is probably not restricted. Bioaccumulation was observed in the invertebrates, and the highest BAFs for Ag in the tissues in relation to the sediment were 15.2 for *P*. *ovata* foot, 11.7 for *P*. *ovata* viscera, and 5.56 for *C*. *nilotica* (SM Table 2). The concentrations in invertebrates were significantly positively correlated with Cu levels (Spearman’s rank test, *r*_s_ = 0.437, *p* < 0.0001 for *C*. *nilotica*; *r*_s_ = 0. 541, *p* = 0.030 for *M*. *bourguignati*; *r*_s_ = 0.786, *p* < 0.0001 and for *P*. *ovata*). Our results therefore point to the potential common sources or intake paths and sequestration for Cu and Ag in the invertebrates. There is a paucity of data on the accumulation of Ag in invertebrates from natural waters. Experimental results, however, demonstrated that under Ag exposure some mollusks can accumulate high levels of Ag in their tissues. For instance, in an experiment where sediment was spiked with AgNO_3_ to 100 mg/kg up to 75 mg/kg dw, Ag was recorded in the hepatopancreas of the viviparid snail *Bellamya aeruginosa* (Bao et al. 2018). This is much higher than recorded in the invertebrates from our study. In freshwater fish, Ag is rapidly taken up through the gills and intestine, and with low trophic transfer, biomagnification does not occur (Wood 2012). We recorded the highest concentrations in *O*. *niloticus* liver; the values varied between the study sites (Kruskal–Wallis test, *X*^2^(4) = 37.232, *p* < 0.0001), with the highest concentrations in fish at the site impacted by Kisumu City (Fig. 2). *Oreochromis niloticus* liver is therefore a potential indicator for Ag contamination. At all sampling sites, the *O*. *niloticus* liver levels were higher than those in the corresponding surface sediment (Fig. 2; SM Table 2). Moreover, the liver levels were 20–119 times higher in *O. niloticus* than in *L. niloticus*. These higher values probably reflect dietary intake and habitat exposure. High concentrations of Ag have been reported in algae and zooplankton (Yoo-iam et al. 2014), which form a significant part of the *O. niloticus* diet. In addition, *O. niloticus* inhabits shallow inshore regions of the lake, where contact with contaminated surface sediment and alternative feeding on detritus may expose it to more Ag. Wood (2012) noted that Ag taken up by fish is sequestered by metallothionein, is well regulated in certain tissues, such as blood, gills, and white muscle by homeostatic mechanisms, and is accumulated in internal organs such as liver and kidney. This implies that beyond the differences in Ag exposure routes between the two fish species, Ag metabolism may differ as well. Homeostatic regulation in the muscle and accumulation in the liver and kidney might explain why Ag was not detected in fish muscle tissue. In the liver of *O*. *niloticus*, Ag concentrations were significantly positively correlated with Cu levels (Spearman’s rank test, *r*_s_ = 0.652, *p* < 0.0001). This positive correlation is strongest at low concentrations of Ag (up to 1.5 mg/kg dw), after which the levels of Cu do not correspond with the rise in Ag levels (Fig. 3). The reasons for these differences are not known yet but certainly should draw attention for future research. Also, the levels of Ni in *O*. *niloticus* liver were positively correlated with Ag (Pearson’s test, *r* = 0.479, *p* = 0.033). Ni uptake may involve proton-coupled divalent metal transporters with binding sites similar to those for Cu and sequestration into Ni-induced metallothioneins (Pyle and Couture 2012). Our results therefore point to potential common sources or intake paths and sequestration for Ni, Cu, and Ag in *O*. *niloticus*. Studies on Ag accumulation in fish are sparse, with very few reports from Africa. Like in our study, no Ag was detected in the muscle tissue of fish from Lake Naivasha, Kenya (Plessl et al. 2017) and Vaal Dam, South Africa (Plessl et al. 2019). In fish liver, mean concentrations of 0.427 ± 0.359 mg/kg dw in *T. zillii* from Lake Naivasha (Plessl et al. 2017) and 1.92 ± 0.83 mg/kg dw in *L. umbratus* from South Africa (Plessl et al. 2019) were reported. These values are higher than our *L. niloticus* liver levels (0.032 ± 0.02 mg/kg dw) but are in the same range as *O. niloticus* liver samples from AB, KK, MN, and RS (Table 1). At KM, however, where the surface sediment was contaminated with Ag, the liver concentrations in *O. niloticus* ranged from 1.03 to 6.90 mg/kg dw with a mean value of 3.45 mg/kg dw, which is higher than in the above-mentioned studies from Lake Naivasha and Vaal Dam, clearly indicating that Ag pollution is reflected in fish liver. The consequences for the fish are still unknown, but we again underline the necessity of more closely examining the development in the distribution of Ag in the environment to avoid long term consequences for fish and other aquatic biota.

### Cd

Aquatic invertebrates are net accumulators of nonessential elements, such as Cd (Abdennour et al. 2000; Rainbow 2007). Cd concentrations in *C*. *nilotica* samples from RS were significantly lower than those from AB and KM (Tukey HSD test, *p* < 0.001). *Mutela bourguignati* from RS also had lower levels compared with MN (Mann–Whitney test, *U* = 13.0, *p* = 0.046). Similarly, we recorded a spatial variation in Cd contents in *P*. *ovata* viscera (one-way ANOVA; *F* (3, 45) = 10.8, *p* < 0.001), with the samples from RS having significantly lower levels than elsewhere in the gulf (Table 3). The lower concentrations in the invertebrate samples from RS corresponded with the lower surface sediment values here; *C*. *nilotica*, *P*. *ovata,* and *M*. *bourguignati* are therefore potential bioindicators of Cd contamination. As indicated in SM (Table 2), the concentrations in *P*. *ovata* viscera and *M*. *bourguignati* were higher than the corresponding levels in the sediment. Cd concentrations in *C*. *nilotica* showed a strong positive correlation with Cu (Pearson’s test, *r* = 0.809, *p* < 0.0001); this points to a common source and/or intake path for these elements. Most of our findings on Cd levels in the invertebrates were not consistent with data from other studies. Mwamburi (2013) did not detect Cd (LOD not stated) in either *C*. *nilotica* or the bivalve *Sphaerium* sp. from Lake Victoria. In our study, Cd concentrations were: 0.046–0.069 mg/kg dw in *C*. *nilotica*, 0.274 and 0.463 mg/kg dw in *M*. *bourguignati*, and 0.60–5.6 mg/kg dw in *P. ovata* viscera. These concentrations, however, were lower than in invertebrates from polluted habitats elsewhere. For instance, 2.4–10.4 mg/kg dw was recorded in the shrimp *A*. *desmarestii* in Algeria (Abdennour et al. 2000) and 5–29 mg/kg dw in the viscera of *Pomacea canaliculata* in Fankou stream (China) (Deng et al. 2008). Regarding food safety for humans, the mean Cd content of *P. ovata* viscera from the AB sampling site (1.11 mg/kg ww) surpassed the 1.0 mg/kg ww safety standard (EU 2008; FAO/WHO 2017), indicating a potential risk for consumption. In freshwater fish, Cd is taken up through gill exposure and is rapidly absorbed by various internal organs from the plasma, with the highest percent absorption occurring in the liver (Mcgeer et al. 2012). In our study, *O. niloticus* liver had higher levels than *L. niloticus* liver (Mann–Whitney test, *U* = 1052, *p* = 0.015). These higher levels in *O. niloticus* may result more from exposure and uptake via the gills from the habitat, namely contaminated shallow inshore areas. Cd has a long biological half-life in vertebrates (Mcgeer et al. 2012), and the levels in the sampling sites may reflect occasional or low-level chronic contamination. This might explain why the highest concentration in fish was recorded in *O. niloticus* liver at RS (Table 4), where Cd was lowest in the surface sediment, leading to a BAF of 2.79. A common source of Cd contamination is runoff from agricultural soils. Cd occurs in phosphate fertilizer to varying degrees and in agricultural soils where phosphate fertilizer is applied (Roberts 2014). In both fish species, no Cd was detected (LOD = 0.005 mg/kg dw) in muscle tissue. In liver tissue, there were significant negative correlations between Cd and Pb concentrations (Spearman’s rank test, *r*_s_ = − 0.337, *p* = 0.007 and *r*_s_ = − 0.370, *p* = 0.011 for *O*. *niloticus* and *L*. *niloticus*, respectively. Contrary to the report by Çoğun et al. (2003), where Cd in *O. niloticus* tissues had no correlation with fish size, the values in *O. niloticus* liver were highly positively correlated with TL (Spearman’s rank test, *r*_s_ = 0.798, *p* < 0.01). In other studies, Otachi et al. (2014) reported mean concentrations of 0.74 mg/kg dw in the muscle and 2.44 mg/kg dw in the liver of *O. leucostictus* from Lake Naivasha. We recorded lower levels in fish tissue. Cd was below 0.005 mg/kg dw in all muscle samples, and the highest mean concentration in liver tissue was 0.333 ± 0.27 mg/kg dw. Based on the data from Otachi et al. (2014) and Outa et al. (2019), the concentrations in surface sediment in Lake Naivasha and Lake Victoria were of the same magnitude; hence, the different Cd contents in the fish can be attributed to site-specific physicochemical conditions and interspecific differences in Cd uptake and metabolism.

### Pb

**Fig. 4:**
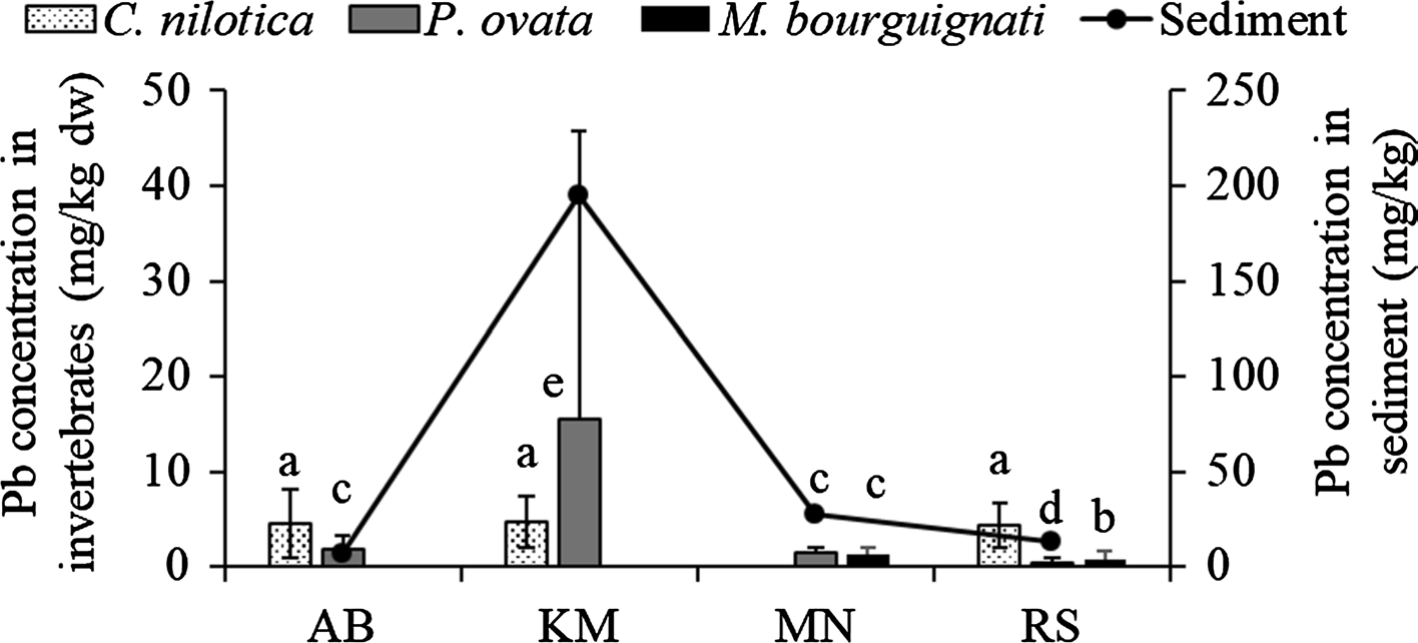
Concentration patterns of Pb in *C*. *nilotica*, *P*. *ovata* viscera, and *M*. *bourguignati* in the current study and the corresponding surface sediment content in different sampling sites (data from Outa et al. 2019)

Pb is a nonessential element whose net accumulation occurs in invertebrates (Abdennour et al. 2000; Rainbow 2007). *Mutela bourguignati* from RS had significantly lower levels of Pb compared with those from MN (Mann–Whitney test, *U* = 13.0, *p* = 0.046). Similarly, the concentrations in *P*. *ovata* viscera varied significantly between the study sites (one-way ANOVA; *F* (3, 45) = 4.087, *p* = 0.012), with the highest values at KM and the lowest at RS (Table 3). These variations corresponded to the Pb levels in the sediment (Fig. 4); accordingly, *M*. *bourguignati* and *P*. *ovata* viscera are potential indicators for Pb contamination. Our results on Pb in the invertebrates differ significantly from reports elsewhere. Abdennour et al. (2000) reported mean concentrations of 11.2–11.5 mg/kg in the atyid shrimp *A*. *desmarestii* in Algeria, which is higher than the mean concentrations (4.38–4.71 mg/kg dw) that we recorded in *C*. *nilotica*. Likewise, concentrations in *M*. *bourguignati* (0.73 ± 0.97 and 1.41 ± 0.68 mg/kg dw) and in *P. ovata* viscera from our study sites (0.48–15.5 mg/kg dw) were lower than the mean in *Sphaerium* sp. (31.8 mg/kg dw) from Lake Victoria (Mwamburi 2013). Morover, Deng et al. (2008) reported much higher levels in viscera of the snail *Pomacea canaliculata* (160–620 mg/kg dw) in Fankou stream, which received effluent from a Zn/Pb mine in southern China. Regarding the implications for human consumption, the Pb content of *P. ovata* viscera (3.09 ± 6.0 mg/kg ww) from the KM sampling site, surpassed the maximum limits of 1 mg/kg ww (EU 2008; FAO/WHO 2017), indicating a risk. In freshwater fish, Pb is taken up from the water into various tissues to varying degrees within and among populations and/or species (Mager 2012). In our study, except for two sites (MN and RS) where concentrations in *O. niloticus* liver were significantly lower (Table 4), Pb tissue concentrations did not show a clear preferential distribution between fish species or in relation to sediment concentration. In both fish species, Pb concentrations were positively correlated with Ni levels in liver tissue (Spearman’s rank test, *r*_s_ = 0.336, *p* = 0.007 and Pearson’s rank test, *r* = 0.686, *p* < 0.0001 for *O*. *niloticus* and *L*. *niloticus*, respectively). Similarly, Pb was positively correlated with Zn in *O*. *niloticus* liver (Spearman’s rank test, *r*_s_ = 0.575, *p* = 0.007) and Cu in *L*. *niloticus* muscle tissue (Spearman’s rank test, *r*_s_ = 0.339, *p* = 0.020). Fish size did not correlate to Pb concentrations in liver and muscle in our study, which agrees with the findings of Szarek-Gwiazda and Amirowicz (2006) and Luczynska et al. (2018). Elsewhere in the region, the mean Pb levels in the muscle and liver of *O*. *niloticus* and *L. niloticus* were at least three times higher than in the corresponding tissues of *O. leucostictus* from Lake Naivasha, where the levels of Pb in the sediment were also lower (Otachi et al. 2014). The levels in muscle tissue (0.012–0.032 mg/kg ww) were, however, below the maximum acceptable limits for human consumption: 0.2 mg/kg ww (EU 2008) and 0.3 mg/kg ww (FAO/WHO 2017) and therefore pose no risk for consumption.

## Conclusions

The invertebrates in this study accumulated nearly all elements measured to higher levels than fish. The exceptions were Cu and Ag, which were highest in *O. niloticus* liver. Contamination of the gulf with trace elements was best mirrored in the invertebrates, which accumulated Cr, Cd, Ag, and Pb corresponding to their levels in the surface sediment. The accumulation in the two fish species and their bioindicative potential corresponded to their habitat characteristics and feeding behavior. The inshore-dwelling, omnivorous *O*. *niloticus*, which is exposed to higher contamination at the littoral areas of the lake, had significantly higher levels of most trace elements and showed a bioindicative potential for Cu and Ag.

Our study shows strong positive correlations between the concentrations of Cu and Ag in the invertebrates and in *O*. *niloticus* in low Ag concentrations pointing to similar pathways for these elements. We also show a negative correlation between fish size and Zn concentrations in muscle and a positive correlation between Cu and Cd in *O*. *niloticus* liver. Regarding the health risk of consuming products from the lake, the levels of Cr, Ni, Cu, Zn, Cd, and Pb in fish muscle do not pose a direct risk. On the other hand, there is potential health risk in relation to the Zn, Cd, and Pb contents of the gastropod *P. ovata* from Winam Gulf. Finally, given that Cr, Ni, and Ag are scarcely monitored in bioaccumulation investigations in African inland water bodies, the data serve as a basis for future investigations, especially for Ag, whose levels were elevated in the liver of the omnivorous, inshore-dwelling fish species.

## Electronic supplementary material

Below is the link to the electronic supplementary material.

Supplementary material 1 (DOCX 19 kb)
