# Supplementary material for: Trace Elements in Crustaceans, Mollusks and Fish in the Kenyan Part of Lake Victoria: Bioaccumulation, Bioindication and Health Risk Analysis
Source: Arch Environ Contam Toxicol. 2020 Feb 4;78(4):589–603. doi: 10.1007/s00244-020-00715-0 (PMC7136317; doi:10.1007/s00244-020-00715-0)
Supplement: Supplementary file 1 — Supplementary material 1 (DOCX 19 kb) [file 244_2020_715_MOESM1_ESM.docx]

**SM Table 1** Total length (TL) and weight of fish samples from the study area: site specific for *O*. *niloticus* and pooled samples for *L*. *niloticus*.

| Fish species | Site | *n* | TL (cm) | | |  | Weight (Kg) | |  |
| --- | --- | --- | --- | --- | --- | --- | --- | --- | --- |
|  |  |  | Mean | SD | Range |  | Mean | SD | Range |
| *O*. *niloticus* | AB | 9 | 15.8 | 2.5 | 12.4 - 20.2 |  | 0.068 | 0.03 | 0.037 - 0.108 |
|  | KM | 20 | 17.4 | 4.9 | 11.2 - 27.3 |  | 0.119 | 0.09 | 0.031 - 0.27 |
|  | KK | 9 | 17.64 | 3.9 | 14.0 - 24.8 |  | 0.131 | 0.09 | 0.061 - 0.322 |
|  | MN | 6 | 17.1 | 2.3 | 14.2 - 20.7 |  | 0.106 | 0.05 | 0.056 - 0.187 |
|  | RS | 20 | 19.6 | 2.8 | 12.4 - 25.0 |  | 0.158 | 0.06 | 0.034 - 0.300 |
| *L*. *niloticus* |  | 46 | 27.2 |  | 16.1 - 55.1 |  | 0.39 | 0.5 | 0.049 - 1.8 |

**SM Table 2** Bioaccumulation factors of selected trace elements in invertebrates and fish tissue from Lake Victoria. Dash (─) indicate where element concentrations were not measured or below LOD in the tissue.

| Element | Site | *C. niloticus*/ sediment | *P. ovata* foot/ sediment | *P. ovata* viscera/ sediment | *M. bourguignati*/ sediment | *O. niloticus* muscle/ sediment | *O. niloticus* liver/ sediment |
| --- | --- | --- | --- | --- | --- | --- | --- |
| Cr | AB | 0.117 | 0.018 | 0.180 | ̶ | 0.005 | 0.011 |
|  | KM | 0.061 | 0.010 | 0.064 | ̶ | 0.002 | 0.006 |
|  | KK | ̶ | ̶ | ̶ | ̶ | 0.003 | 0.008 |
|  | MN | ̶ | 0.010 | 0.080 | 0.140 | 0.014 | 0.010 |
|  | RS | 0.045 | 0.022 | 0.092 | 0.141 | 0.004 | 0.014 |
|  |  |  |  |  |  |  |  |
|  |  |  |  |  |  |  |  |
| Ni | AB | 0.162 | 0.091 | 2.27 | ̶ | 0.003 | 0.012 |
|  | KM | 0.079 | 0.108 | 0.775 | ̶ | ̶ | 0.012 |
|  | KK | ̶ | ̶ | ̶ | ̶ | ̶ | 0.010 |
|  | MN | ̶ | 0.011 | 0.692 | 0.167 | ̶ | 0.006 |
|  | RS | 0.046 | 0.051 | 0.556 | 0.076 | 0.004 | 0.010 |
|  |  |  |  |  |  |  |  |
| Cu | AB | 5.26 | 2.60 | 2.05 | ̶ | 0.023 | 8.54 |
|  | KM | 1.92 | 1.79 | 2.25 | ̶ | 0.035 | 5.32 |
|  | KK | ̶ | ̶ | ̶ | ̶ | 0.026 | 15.8 |
|  | MN | ̶ | 3.96 | 4.40 | 0.384 | 0.035 | 7.50 |
|  | RS | 1.65 | 2.39 | 1.93 | 0.138 | 0.015 | 3.62 |
|  |  |  |  |  |  |  |  |
| Zn | AB | 0.854 | 0.804 | 16.5 | ̶ | 0.286 | 1.59 |
|  | KM | 0.247 | 0.184 | 3.03 | ̶ | 0.078 | 0.334 |
|  | KK | ̶ | ̶ | ̶ | ̶ | 0.160 | 1.18 |
|  | MN | ̶ | 0.473 | 6.07 | 7.89 | 0.220 | 0.853 |
|  | RS | 0.772 | 0.653 | 14.5 | 4.08 | 0.207 | 0.931 |
|  |  |  |  |  |  |  |  |
| As | AB | 0.308 | 0.103 | 0.185 | ̶ | ̶ | ̶ |
|  | KM | 0.172 | 0.144 | 0.125 | ̶ | ̶ | ̶ |
|  | KK | ̶ | ̶ | ̶ | ̶ | ̶ | ̶ |
|  | MN | ̶ | 0.065 | 0.059 | 0.020 | ̶ | ̶ |
|  | RS | 0.804 | 0.312 | 0.164 | 0.076 | ̶ | 0.030 |
|  |  |  |  |  |  |  |  |
| Sr | AB | 14.7 | 2.82 | 2.58 | ̶ | ̶ | ̶ |
|  | KM | 10.7 | 3.27 | 4.29 | ̶ | ̶ | ̶ |
|  | KK | ̶ | ̶ | ̶ | ̶ | ̶ | ̶ |
|  | MN | ̶ | 0.34 | 0.39 | 1.11 | ̶ | 0.008 |
|  | RS | 1.92 | 0.48 | 1.10 | 3.11 | ̶ | ̶ |
|  |  |  |  |  |  |  |  |
| Ag | AB | 5.56 | 6.70 | 3.89 | ̶ | ̶ | 15.3 |
|  | KM | 0.310 | 0.49 | 0.523 | ̶ | ̶ | 2.54 |
|  | KK | ̶ | ̶ | ̶ | ̶ | ̶ | 20.6 |
|  | MN | ̶ | 8.11 | 9.61 | 0.943 | ̶ | 15.8 |
|  | RS | 4.12 | 15.2 | 11.7 | 0.895 | ̶ | 28.2 |
|  |  |  |  |  |  |  |  |
| Cd | AB | 0.360 | 0.923 | 28.9 | ̶ | ̶ | 0.322 |
|  | KM | 0.121 | 0.107 | 4.51 | ̶ | ̶ | 0.355 |
|  | KK | ̶ | ̶ | ̶ | ̶ | ̶ | 0.741 |
|  | MN | ̶ | 0.119 | 4.48 | 1.58 | ̶ | 0.198 |
|  | RS | 0.388 | 0.216 | 5.02 | 2.30 | ̶ | 2.785 |
|  |  |  |  |  |  |  |  |
| Pb | AB | 0.670 | 0.041 | 0.273 | ̶ | 0.023 | 0.072 |
|  | KM | 0.024 | 0.004 | 0.079 | ̶ | 0.001 | 0.003 |
|  | KK | ̶ | ̶ | ̶ | ̶ | 0.008 | 0.032 |
|  | MN | ̶ | 0.006 | 0.056 | 0.051 | 0.003 | 0.003 |
|  | RS | 0.328 | 0.015 | 0.036 | 0.055 | 0.008 | 0.009 |

BAFs calculated based on element concentrations in water and sediment data from Outa et al. 2019
